# The Effectiveness of Microcurrent Stimulation Combined with Sound Therapy for Tinnitus Relief: A Preliminary Study

**DOI:** 10.3390/audiolres14010012

**Published:** 2024-01-29

**Authors:** Donghyeok Lee, Youngchan Jeong, Sumin Lee, Tae-Jun Jin, In-Ki Jin

**Affiliations:** 1 Department of Speech Pathology and Audiology, Graduate School, Hallym University, Chuncheon-si 24252, Gangwon-do, Republic of Korea; deggw0918@gmail.com (D.L.); youngchanj@naver.com (Y.J.); eaeno123@gmail.com (S.L.); jtj73872711@gmail.com (T.-J.J.); 2 Division of Speech Pathology and Audiology, Research Institute of Audiology and Speech Pathology, Hallym University, Chuncheon-si 24252, Gangwon-do, Republic of Korea

**Keywords:** microcurrent stimulation, neuroplasticity, rehabilitation, sound therapy, tinnitus

## Abstract

Various stimulation-based rehabilitation approaches have been proposed to alleviate tinnitus. This study aimed to determine the efficacy of a rehabilitation approach that simultaneously provides microcurrent and sound stimulation for tinnitus relief. Twenty-eight participants with chronic sensorineural tinnitus were randomly assigned to one of two groups based on the rehabilitation approaches (sound therapy-only group and combined microcurrent and sound therapy group). Each participant underwent sound therapy or simultaneous stimulation for approximately 2 h daily for 3 months. The effectiveness of the rehabilitation approaches was determined based on changes in the Korean version of the tinnitus primary function questionnaire (K-TPFQ) and visual analog scale for loudness (VAS-L) scores at baseline, 1.5 months, and 3 months. For the K-TPFQ scores, both groups exhibited a large effect of rehabilitation; however, for the VAS-L scores, the simultaneous stimulation group demonstrated a large effect of rehabilitation, whereas the sound therapy group exhibited a small effect. Therefore, a rehabilitation approach that combines sound stimulation with microcurrent stimulation can improve response and perception in tinnitus.

## 1. Introduction

Tinnitus is defined as the perception of sound independent of external sound stimuli [1]. Tinnitus is prevalent in approximately 10–15% of the adult population, with approximately 2% of patients reporting a significant reduction in quality of life [2,3]. Common difficulties reported by patients with tinnitus include sleep disturbances, difficulty concentrating, and emotional difficulties such as depression and irritability [2,4]. Many subtypes of tinnitus exist, and choosing an appropriate treatment based on the cause and symptoms is important [5,6].

Sound therapy is a typical rehabilitation approach for patients with chronic tinnitus, aiming to habituate patients to tinnitus and reduce focus on it [7,8,9]. In a study by Jin et al. (2022), 14 of 38 participants with chronic tinnitus who received sound therapy for at least 3 h daily for 3 months reported significant tinnitus relief [9]. In another study, 5 of 15 participants with chronic tinnitus who received sound therapy reported significant relief from tinnitus [8]. Sound therapy does not appear to be effective for all patients with chronic tinnitus; however, many studies have reported significant reductions in tinnitus or habituation to tinnitus in some participants who have undergone sound therapy [10,11,12].

Researchers have proposed a rehabilitation approach that combines electrical and sound stimulation to improve the effectiveness of tinnitus rehabilitation [13,14]. Electrical stimulation approaches are known to be safe methods used primarily to reduce pain. Various modalities of electrical stimulation approaches have been used in studies on tinnitus relief since transcutaneous electrical nerve stimulation (TENS) of the median nerve was first demonstrated to modulate tinnitus perception in some patients [15]. Many theories have been proposed regarding the mechanism of tinnitus, one of which suggests that tinnitus results from hyperactive neuronal activity in the central auditory system [16]. Therefore, tinnitus can be attributed to spontaneous maladaptive neuroplasticity [17]. The mechanism of the simultaneous approach of sound and electrical stimulation is as follows: sound stimulation selectively activates auditory nerve responses in patients with tinnitus based on the frequency characteristics of the sound. This neural response is further activated through electrical stimulation. Therefore, activating frequency regions other than the already activated tinnitus frequency regions can suppress prominent tinnitus responses. Coactivation through repeated and sustained sound and electrical stimulation can alter maladaptive neuroplasticity.

Tyler et al. [13] evaluated the effects of vagus nerve stimulation paired with sound in patients with chronic tinnitus. Surgical intervention delivered electrical stimulation directly to the vagus nerve, whereas sound stimulation was delivered through headphones. Each vagus nerve stimulation consisted of 15 0.8-mA constant-current charge-balanced pulses, and the sound stimuli consisted of tones that stimulated a frequency range outside the participant’s tinnitus frequency. The participants underwent simultaneous stimulation rehabilitation for 2.5 h daily for 6 weeks. Consequently, 50% (8/16) of the participants demonstrated clinically meaningful improvement based on changes in the tinnitus questionnaire scores after 6 weeks. Conlon et al. [14] measured tinnitus relief using a rehabilitation method that simultaneously provided sound and electrical stimulation through the ears and tongue, respectively. The participants completed two 30 min treatment sessions daily for 12 weeks. The researchers reported a significant reduction in the mean tinnitus questionnaire score. None of the studies reported any serious adverse events [13,14].

Several studies have reported the promise of rehabilitation approaches that combine sound and electrical stimulation to relieve tinnitus; however, some areas require further research [13,14]. Whether rehabilitation combining sound and electrical stimulation is more effective than traditional sound therapy remains unclear. Studies on sound therapy alone have also reported significant tinnitus relief in 30–50% of participants when assessing individualized tinnitus relief [9,11]. However, studies on sound therapy alone and those combining sound and electrical stimulation are not directly comparable because of differences in participant characteristics, rehabilitation duration, and assessment methods. Therefore, a study comparing tinnitus relief in a rehabilitation group that receives simultaneous sound and electrical stimulation while controlling for other variables to that in a group that receives sound stimulation alone could help confirm the validity of a rehabilitation approach that provides both sound and electrical stimulation.

Another consideration is the location of electrical stimulation, which may require an evaluation of its effectiveness in multiple areas. Sound and electrical co-stimulation studies have been conducted on invasive stimulation via the vagus nerve and non-invasive stimulation via the tongue; however, various body sites can stimulate brain regions, including the auditory or central nervous systems. Therefore, studies on the effectiveness of electrical stimulation for tinnitus relief at different body sites are required to evaluate the efficacy of rehabilitation approaches that provide both sound and electrical stimulation.

This study aimed to determine the efficacy of a rehabilitation approach that simultaneously provides non-invasive microcurrent and sound stimulation to the periphery of the ear for tinnitus relief. This study provides additional information on the implementation of a rehabilitation approach that involves simultaneous sound and electrical stimulation to alleviate tinnitus. Simultaneous stimulation therapy may be an effective option for patients with tinnitus who require tinnitus suppression and improvement in the tinnitus response.

## 2. Materials and Methods

### 2.1. Participants

The participants in this study were screened following the inclusion and exclusion criteria based on previous research on sound therapy and microcurrent stimulation for tinnitus interventions [13,14,18]. Individuals who (1) had been diagnosed with sensorineural tinnitus without signs of somatosensory tinnitus by an otolaryngologist, (2) were adults aged ≥ 20 years or older, (3) had experienced tinnitus for at least 6 months, and (4) complained of discomfort and irritation owing to tinnitus were included in this study. Individuals who (1) had a hearing threshold of ≥70 dB, with an average threshold of 500, 1000, and 2000 Hz on the better ear, (2) had moderate-to-severe dementia as indicated by a score of <20 on the Mini-Mental State Examination (MMSE), (3) had other electrical stimulators implanted (for example, cochlear implants and pacemakers), (4) were taking neuropsychiatric medications or medications known to worsen tinnitus, (5) had symptoms of Meniere’s disease, posterior fossa disease, or active middle ear disease (such as inflammation, pus, and swelling), (6) had a history of central nervous system disorders such as hallucinations, (7) were currently receiving tinnitus treatment from other institutions, and (8) were involved in a lawsuit related to tinnitus were excluded from the study.

**Figure 1:**
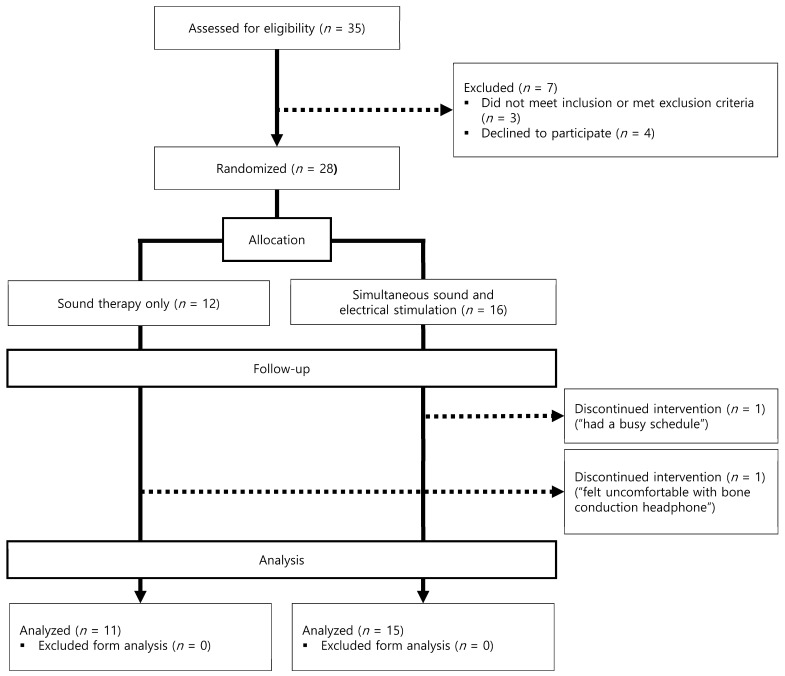
Flowchart of the number of participants for each study phase.

Figure 1 shows a flowchart of the number of participants in each study phase. Thirty-five participants were recruited for the study. A total of 9 individuals dropped out of the study, 7 at baseline and 2 at 1.5 months, leaving 26 participants. Three applicants were excluded because they met the following exclusion criteria: (1) a hearing threshold of >75 dB hearing loss for all frequencies measured (*n* = 2) and (2) using neurological medications (*n* = 1). Four applicants declined to participate in the study for the following reasons: (1) discomfort while hearing the notched noise stimulus (*n* = 1) and (2) having a busy schedule (*n* = 3). Two patients met the inclusion criteria and participated in the study but dropped out owing to discomfort with the bone-conduction headphones (*n* = 1) and busy schedules (*n* = 1). The study analyzed data from 26 participants (sound therapy group: 11; simultaneous stimulation group: 15).

### 2.2. Interventions

The participants were randomly assigned to either the sound therapy group (control group) or simultaneous stimulation group (experimental group) based on the treatment type for 3 months. However, when conducting the randomized group assignments, we weighted the experimental group by approximately 15% to ensure that more people were assigned to the experimental group than to the control group. Randomization was performed using the randomization function in Microsoft Excel (Microsoft, Redmond, WA, USA). Four qualified audiologists with a master’s degree conducted this study.

The sound therapy group was instructed to undergo 2 h of notched noise sound therapy daily using bone-conduction headphones (Etereo Bm+, MIJ Co., Chuncheon, Republic of Korea), with the stimulus presented at a mixing point. The mixing point refers to the level at which the sound therapy stimulus blends with the tinnitus sound level without masking or obscuring it [19]. This study used sound therapy stimuli reported in previous studies to provide effective tinnitus relief to some participants [18]. Briefly, notched noise was created by applying a band-reject filter with a bandwidth of 1/2 octave centered at the tinnitus frequency to the broadband noise (white noise ranging from 100 to 22,050 Hz). The slope of the band-rejection filter was 300 dB/octave. Subsequently, the frequency bandwidths of 3/8 octaves on each notch side were amplified by 20 dB [18]. The notched stimuli presented broadband noise with tinnitus frequencies filtered out as sound stimuli, inducing vigorous neural response activity in neural regions outside the tinnitus frequencies. Through lateral suppression, notched stimulation sound therapy induces a balance between the frequency region where abnormal neural activity occurs (tinnitus frequency) and other frequency regions, leading to a lower perception of tinnitus [20].

**Figure 2:**
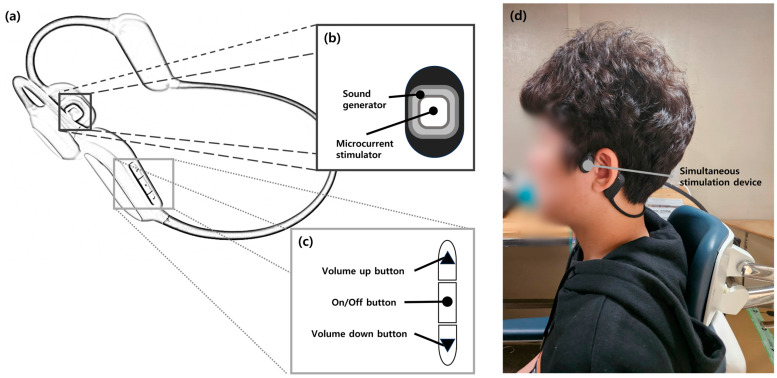
A schematic of the simultaneous stimulation device (**a**), stimulator (**b**), control button (**c**), and a photo of wearing the simultaneous stimulation device (**d**).

The simultaneous stimulation group underwent notched stimulation sound therapy in addition to microcurrent stimulation. The notched stimulation was identical to that used in the sound therapy group. The simultaneous stimulation device consisted of two microcurrent stimulators, two sound generators, and control buttons (Figure 2). The output values of the microcurrent generators were developed to conform to the standards of the “Guideline for Safety and Performance Evaluation of Personal Electrical Stimulator” issued by the Korean Ministry of Food and Drug Safety (maximum current density < 2 mA/cm^2^, maximum average power density < 0.25 W/cm^2^) [21]. Microcurrents were delivered simultaneously with a sound therapy stimulus through a microcurrent stimulator in two positions, left and right, and the stimulation intensity was divided into 10 levels with intensities of <2 mA/cm^2^. The intervention was performed using a microcurrent device for 30 min per session, no more than four times a day. The researchers set the stimulation level to provide microcurrent stimulation one level below the level at which each participant began to perceive microcurrents. The participants were also instructed to rest for at least 1 h between treatments to ensure safety.

### 2.3. Measurements

Outcome measures were based on the following validated tools to assess the degree of tinnitus relief: (1) the Korean version of the tinnitus primary function questionnaire (K-TPFQ) and (2) visual analog scale for loudness (VAS-L) [22,23]. The K-TPFQ is a tinnitus questionnaire that measures secondary difficulties (such as emotions, hearing, concentration, and sleep) caused by tinnitus on a scale of 0 to 100, with higher scores indicating greater difficulties caused by tinnitus. The VAS-L requires participants to rate the magnitude of their perceived tinnitus on a scale of 0 to 100, with higher scores indicating greater perceived tinnitus.

### 2.4. Procedure

**Figure 3:**
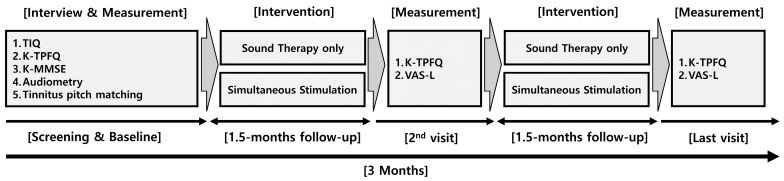
A protocol diagram of the study procedure. K-TPFQ, Korean version of the tinnitus primary function questionnaire; TIQ, tinnitus intake questionnaire; VAS-L, visual analog scale for loudness.

Figure 3 illustrates the protocol used in this study. This study was conducted at the Research Institute of Audiology and Speech Pathology at Hallym University. At baseline, questionnaires and air conduction audiometry were administered to confirm eligibility based on the inclusion and exclusion criteria. Three types of questionnaires were used for screening: (1) the tinnitus intake questionnaire (TIQ), (2) K-TPFQ, and (3) Korean version of the MMSE (K-MMSE). The TIQ confirmed the applicant’s demographic and tinnitus information, including the VAS-L. The K-MMSE is a tool for assessing cognitive function that has been extensively employed in clinical assessments and research studies involving individuals with dementia. [24,25] A K-MMSE score of <20 indicates moderate to severe dementia.

Audiometry was performed at 500, 1000, and 2000 Hz for screening. Pitch matching was then performed to identify the customized notch sound stimuli for each participant [26]. We used narrowband pulses with a range of frequency energies to present two pulsed stimuli with distant frequency specificity and asked each participant to choose a stimulus sound similar to their tinnitus. Once each participant selected one of the two pulsed stimuli, the pulsed stimuli around that stimulus were presented and measured until each participant identified the stimulus they responded to as most similar to their tinnitus frequency region. The pitch matching and audiometry were performed using a double-walled sound booth (GSI 61; Grason-Stadler, Eden Prairie, MN, USA).

After all the measurements were completed, each participant was randomly assigned to either the sound therapy group or the simultaneous stimulation group. The researchers then provided the participants devices tuned to their tinnitus sounds and trained them on how to use them. At each step, candidates who did not meet the criteria or declined to participate in the study were excluded from data collection. The intervention was conducted for 3 months, with follow-ups at 1.5 and 3 months. The researchers sent the participants text messages encouraging them to use their devices during the study and visited the participants’ houses to help if they encountered any study-related issues. At follow-up, two types of questionnaires were administered to measure changes in the participants’ tinnitus: (1) the K-TPFQ and (2) VAS-L.

### 2.5. Statistical Analyses

Baseline comparisons of the mean age, tinnitus duration, K-TPFQ scores, and VAS-L scores between the groups were performed using an independent sample *t*-test. To identify interactions between the two groups (sound therapy group and simultaneous stimulation group) and the three time points (baseline, 1.5-month follow-up, and 3-month follow-up) in terms of the K-TPFQ and VAS-L scores, repeated measures analysis of variance was used. Statistical significance was set at an α of 0.05. Bonferroni correction was used to identify the significance of the change in scores across time points in each group. Additionally, we analyzed the change in scores on the measurement tools from baseline to 3 months using Cohen’s d to determine the effect size for each group. All statistical analyses were performed using SPSS software (version 26.0; SPSS Inc., Chicago, IL, USA).

We utilized the minimum clinically important difference (MCID) value to assess the inter-individual validity of rehabilitation. The MCID represents the smallest change in score that is considered meaningful [27]. The suggested MCIDs of the K-TPFQ and VAS-L scores are 13 and 15 points, respectively [23,28]. The treatment effect was considered clinically significant when the follow-up value of the questionnaire was above the MCID.

## 3. Results

The results of the comparison between the two groups at baseline are presented in Table 1. In the sound therapy group, the mean age was 74 years, and the mean duration of tinnitus was 130.36 months. The measured tinnitus frequency was 1000 Hz for three participants, 4000 Hz for five, and 8000 Hz for three. The mean K-TPFQ score was 42.34, and the mean VAS-L score was 42.73. In the simultaneous stimulation group, the mean age was 70.87 years, and the mean duration of tinnitus was 155.73 months. The measured tinnitus frequency was 2000 Hz for three participants, 4000 Hz for seven, and 8000 Hz for five. The mean K-TPFQ score was 43.97, and the mean VAS-L score was 54.33. At baseline, no significant differences existed in the mean age, tinnitus duration, and K-TPFQ and VAS-L scores between the groups (*p* > 0.05).

**Figure 4:**
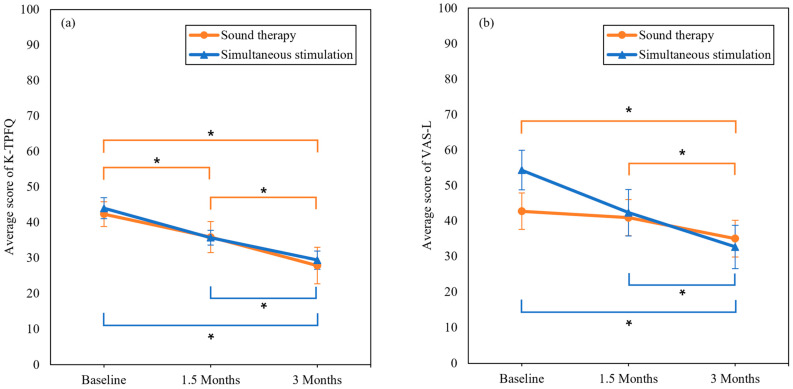
Mean scores and standard errors of the K-TPFQ (**a**) and VAS-L scores (**b**) for the two groups at baseline, 1.5 months, and 3 months. Asterisks are used to indicate statistically significant results. K-TPFQ, Korean version of the tinnitus primary function questionnaire; VAS-L, visual analog scale for loudness.

In both groups, the mean K-TPFQ scores decreased significantly over time (Figure 4). In the sound therapy group, the mean TPFQ scores decreased to 42.34 (standard deviation [SD]: 9.82, standard error [SE]: 2.96) at baseline, 35.82 (SD: 6.97, SE: 2.10) at 1.5 months, and 27.82 (SD: 8.45, SE: 2.55) at 3 months (*F* = 17.116, *p* = 0.001). The change from baseline to 1.5 months was significant (*p* = 0.021); the decrease from 1.5 months to 3 months was also significant (*p* = 0.005). In the simultaneous stimulation group, the mean TPFQ scores decreased to 43.97 (SD: 13.53, SE: 3.49) at baseline, 35.67 (SD: 16.95, SE: 4.38) at 1.5 months, and 29.35 (SD: 19.74, SE: 5.10) at 3 months (*F* = 10.421, *p* = 0.0001). The change from baseline to 1.5 months was insignificant (*p* = 0.101), whereas the decrease from 1.5 months to 3 months was significant (*p* = 0.031). The effect size was large for both the sound therapy (*d* = 0.93) and simultaneous stimulation (*d* = 0.86) groups.

For both groups, the mean VAS-L scores significantly decreased over time (Figure 4). In the sound therapy group, the mean VAS-L scores decreased to 42.73 (SD: 16.94, SE: 5.11) at baseline, 40.91 (SD: 16.86, SE: 5.08) at 1.5 months, and 35.00 (SD: 17.18, SE: 5.18) at 3 months (*F* = 6.85, *p* = 0.005). The change from baseline to 1.5 months was insignificant (*p* = 1.000), whereas the decrease from 1.5 months to 3 months was significant (*p* = 0.032). In the simultaneous stimulation group, the mean VAS-L scores decreased to 54.33 (SD: 21.70, SE: 5.60) at baseline, 42.33 (SD: 25.42, SE: 6.56) at 1.5 months, and 32.67 (SD: 23.67, SE: 6.11) at 3 months (*F* = 13.878, *p* = 0.001). The change from baseline to 1.5 months was insignificant (*p* = 0.064), whereas the decrease from 1.5 months to 3 months was significant (*p* = 0.004). The sound therapy group exhibited a small effect size (*d* = 0.45), whereas the simultaneous stimulation group exhibited a large effect size (*d* = 0.95).

**Figure 5:**
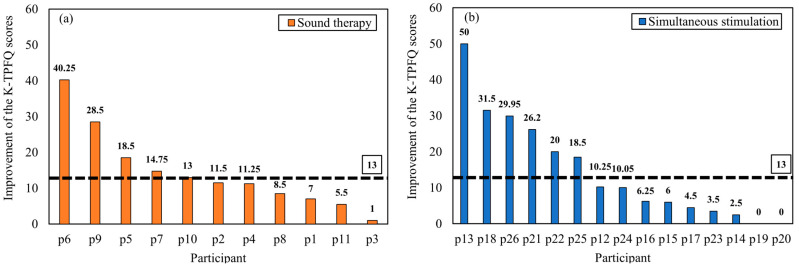
Improvements in the K-TPFQ scores in the sound therapy group (**a**) and simultaneous stimulation group (**b**). The dotted black lines indicate the MCID value. MCID, minimum clinically important difference; K-TPFQ, Korean version of the tinnitus primary function questionnaire.

**Figure 6:**
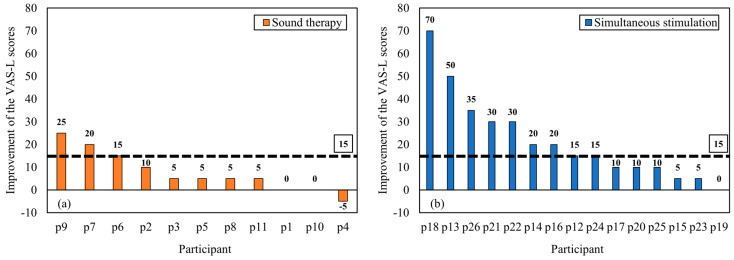
Improvements in the VAS-L scores in the sound therapy group (**a**) and simultaneous stimulation group (**b**). The dotted black lines indicate the MCID value. MCID, minimum clinically important difference; VAS-L, visual analog scale for loudness.

The extent of individual tinnitus improvement was analyzed based on the MCID values of the K-TPFQ (13 points) and VAS-L scores (15 points). Figure 5 illustrates individual K-TPFQ score changes between baseline and 3 months. For the K-TPFQ scores, 5 of 11 (45.45%) patients in the sound therapy group exhibited an improvement of ≥13 points, and 6 of 15 (40.00%) patients in the simultaneous stimulation group exhibited an improvement of ≥13 points. Figure 6 illustrates individual VAS-L score changes between baseline and 3 months. For the VAS-L scores, 3 of 11 (27.27%) patients in the sound therapy group had an improvement of ≥15 points, whereas 9 of 15 (60.00%) patients in the simultaneous stimulation group exhibited an improvement of ≥15 points.

## 4. Discussion

We determined the effectiveness of a tinnitus rehabilitation method that simultaneously involves sound therapy and microcurrent stimulation (simultaneous stimulation). We divided the tinnitus rehabilitation group into a simultaneous stimulation group (experimental group) and sound therapy group (control group) for 3 months. The K-TPFQ was used to measure the improvement in quality of life based on tinnitus. The VAS-L was used to measure the change in the loudness of tinnitus. The statistical analysis was based on average values by group, and the study results revealed a significant improvement in quality of life and reduction in tinnitus loudness in both groups.

One of the two research questions of this study was to determine whether the simultaneous stimulation approach is more effective than sound therapy alone in relieving tinnitus. First, the effectiveness of sound therapy in this study was measured by the change in the K-TPFQ score, with 45.5% (5/11) of participants exhibiting significant improvement. This finding is similar to that of previous studies that reported that approximately 30–50% of participants exhibited improvement in the tinnitus questionnaire score with sound therapy alone [8,9]. Analyses based on mean values by group revealed significant improvements in the K-TPFQ and VAS-L scores in both groups; however, effect size comparisons revealed differences between the groups. When analyzed by group effect size, both groups exhibited a large effect on the K-TPFQ. However, for the VAS-L, the simultaneous stimulation group exhibited a large effect, whereas the sound therapy group exhibited a small effect. These differences in the effect size results by group may be related to improvements in individual VAS-L scores. Compared with 9 of 15 (60.0%) participants in the simultaneous stimulation group, only 3 of 11 (27.3%) participants in the sound therapy group exhibited improvement in their VAS-L scores above the MCID values. These findings suggest that simultaneous stimulation may be more effective than sound therapy alone for reducing the perceived loudness of tinnitus.

Of the 26 participants in the study, 2 in the simultaneous stimulation group reported that their tinnitus had completely disappeared after participating in the study. The two participants (P13 and P18) were women, and their ages were 85 (P13) and 74 (P18) years, respectively. The duration of P13’s tinnitus was 3 years, and their K-TPFQ and VAS-L scores improved from 50 (baseline) to 0 (3 months). The duration of P18’s tinnitus was 15 years, their K-TPFQ score improved from 31.5 (baseline) to 0 (3 months), and the VAS-L score improved from 70 (baseline) to 0 (3 months). Both participants reported that the pitch-matching results were measured at 4000 Hz and that the tinnitus sound was consistent. Additionally, they consistently underwent simultaneous stimulation therapy for 2 h daily during the study period. Many reasons may explain why tinnitus disappeared completely in these two participants; the presentation of a steady sound and microcurrent stimulation may have activated neuroplasticity to suppress maladaptive spontaneous neuroplasticity [17]. The notched sound therapy stimuli may have effectively stimulated areas outside the participants’ tinnitus frequency, especially because the participants’ tinnitus frequency was constant (4000 Hz) and self-reported as constant [20]. The results of this study suggest that accurately measuring an individual’s tinnitus frequency may be important to presenting an optimized notched sound stimulus; however, further research is needed.

The second research question of this study was to determine an effective location for electrical stimulation apart from the body parts that have been presented in previous studies. Previous studies have reported tinnitus relief from microcurrent stimulation rehabilitation combined with sound therapy via invasive vagus nerve or non-invasive tongue stimulation [13,14]. In this study, microcurrent stimulation was applied to the bone around the ear. The study revealed that the K-TPFQ scores improved in 40% (6/15) of the participants, and the VAS-L scores improved significantly in 60% (9/15). This appears to be comparable to that in the study by Tyler et al. (2017) [13], who reported that the tinnitus questionnaire scores significantly improved in 50% (8/16) of participants who received invasive vagus nerve stimulation. As periauricular stimulation carries signals to the brain via the auditory, trigeminal, and facial nerves, microcurrent stimulation through the skin and bone around the ear may be transmitted to the brain, activating plasticity to suppress tinnitus [29]. Therefore, the results of this study suggest that non-invasive microcurrent stimulation around the ear may be one of the effective routes of stimulation delivery for tinnitus suppression.

This study revealed that simultaneous stimulation therapy, combining microcurrent and sound stimulation, may effectively alleviate and suppress tinnitus. However, it has some limitations. First, this study confirmed that simultaneous stimulation therapy through the bones around the ear may be more effective in reducing tinnitus perception than sound therapy alone; however, it could not establish its superiority over other microcurrent methods. Some studies have suggested that invasive stimulation methods may be more effective in suppressing tinnitus through deep brain stimulation than non-invasive stimulation, such as skin or muscle stimulation; however, direct comparative studies are limited [13,29]. Therefore, comparative studies between non-invasive and invasive stimulation may be needed in the future. Second, we could not confirm the effective stimulation levels from the results of this study. For safety considerations, the current stimulation level was set to be one level lower than the level at which each participant began to perceive the current stimulation. However, if deep brain stimulation is more effective in suppressing tinnitus, the effect of tinnitus suppression may be greater at a higher current level than that applied in this study. Therefore, follow-up studies are necessary to confirm the effective electrical stimulation levels. In this study, participants with an average pure tone threshold of a less than 70 dB hearing level (HL) were selected for participation to avoid the necessity for a stimulus loudness that would be too loud for participants with hearing thresholds above 70 dB HL when performing sound therapy. However, the hearing threshold criteria applied in this study may be somewhat broad. Different hearing threshold levels of participants may affect the response to tinnitus and the effectiveness of tinnitus rehabilitation. Therefore, future studies should consider differences in the effectiveness of simultaneous stimulation treatments in groups with different hearing threshold levels. In addition, this study did not measure participants’ high-frequency hearing thresholds (4 or 8 kHz); hence, given the high correlation between high-frequency hearing thresholds and tinnitus in tinnitus patients, future studies should consider assessing high-frequency hearing thresholds [30]. Finally, this was a preliminary study, and the number of participants was small. Therefore, follow-up studies with larger numbers of patients are required to further validate the results of this study.

## 5. Conclusions

The results of this study reveal that simultaneous stimulation therapy, which combines sound and microcurrent stimulation, can suppress tinnitus and improve its response. Therefore, simultaneous stimulation therapy may be an effective option for patients with tinnitus who require tinnitus suppression and improvement in the tinnitus response.

## Figures and Tables

**Table 1:** Participants’ demographic information, tinnitus characteristics, and K-TPFQ and VAS-L scores at baseline.

		Sound Therapy Group (*n* = 11)	Simultaneous Stimulation Group (*n* = 15)	Independent *t*-Test	
				*t*-Value	*p*-Value
Sex	Male	5	7	N/A	
	Female	6	8		
Age (years)	Mean (SD)	74 (5.87)	70.87 (9.94)	−0.925	0.364
	Range	65–83	47–85		
Tinnitus duration (months)	Mean (SD)	130.36 (133.14)	155.73 (135.26)	0.476	0.639
	Range	24–480	8–360		
Tinnitus pitch	Frequency in Hz (*n*)	1000 (3), 4000 (5), 8000 (3)	2000 (3), 4000 (7), 8000 (5)	N/A	
K-TPFQ score	Mean (SD)	42.34 (9.82)	43.97 (13.53)	0.338	0.738
	Range	30–74.5	31.5–66.5		
VAS-L score	Mean (SD)	42.73 (16.94)	54.33 (21.70)	1.473	0.154
	Range	20–65	20–90		

*n*: number of participants; Hz, Hertz; K-TPFQ, Korean version of the tinnitus primary function questionnaire; SD, standard deviation; VAS-L, visual analog scale for loudness.

## Data Availability

The data used in this study are available in Excel upon request.
